# Comparison of the Clinical Performance of Refractive Rotationally Asymmetric Multifocal IOLs with Other Types of IOLs: A Meta-Analysis

**DOI:** 10.1155/2018/4728258

**Published:** 2018-09-27

**Authors:** Zequan Xu, Wenzhe Li, Lianqun Wu, Shuang Xue, Xu Chen, Qiang Wu

**Affiliations:** ^1^Department of Ophthalmology, Shanghai Jiao Tong University Affiliated Sixth People's Hospital, No. 600, Yishan Road, Xuhui District, Shanghai 200233, China; ^2^Clinical Medical College, Tianjin Medical University, No. 176, Xueyuan Road, Dagang District, Tianjin 100270, China; ^3^Department of Ophthalmology, Changzheng Hospital, Second Military Medical University, 415 Fengyang Road, Shanghai, 200003 Shanghai, China; ^4^Department of Ophthalmology, People's Hospital of Hegang, No. 1, Dianxin Road, Gongnong District, Hegang 154100, Heilongjiang, China; ^5^Department of Cataract and Glaucoma, Shanghai Aier Eye Hospital, No. 1286, Hongqiao Road, Shanghai 200336, China

## Abstract

**Objective:**

To compare the clinical performance of refractive rotationally asymmetric multifocal intraocular lens (IOLs) with spherical monofocal, accommodating, and bifocal IOLs.

**Methods:**

A comprehensive literature search of PubMed, EMBASE, Cochrane Controlled Trials Register, and Web of Science up to February 2017 was performed to identify randomized controlled trials (RCT) and comparative cohort studies. Main outcomes were uncorrected distance visual acuity (UDVA), uncorrected intermediate visual acuity (UIVA), uncorrected near visual acuity (UNVA), higher-order aberrations (HOAs), MTF, Strehl ratio, and residual sphere and cylinder.

**Results:**

Mplus provided significantly worse UDVA than spherical monofocal IOLs (WMD: 0.13, *P*=0.008), but significantly better UIVA than high-add bifocal IOLs (WMD: −0.19, *P* < 0.00001), spherical monofocal IOLs (WMD: −0.12, *P* < 0.0001), and accommodating IOLs (WMD: −0.21, *P* < 0.00001). Mplus provided significantly worse UNVA than high-add bifocal IOLs (WMD: 0.07, *P* < 0.00001), but significantly better UNVA than spherical monofocal IOLs (WMD: −0.19, *P* < 0.00001). Mplus resulted in significantly higher HOAs than high-add bifocal IOLs (WMD: 0.38, *P* < 0.00001) and spherical monofocal IOLs (WMD: 0.51, *P*=0.0004). Mplus provided a significantly lower MTF cut-off and Strehl ratio than other type of IOLs.

**Conclusion:**

The Mplus IOLs perform best regarding intermediate visual acuity whereas they lack in distance visual acuity compared to monofocal IOLs and near visual acuity compared to bifocal IOLs. These results may be due to structure of Mplus IOLs resulting in higher-order aberrations.

## 1. Introduction

Due to the popularity and availability of premium intraocular lens (IOLs), the main goal of cataract surgery has shifted from sight rehabilitation to restoring vision at as many distances as possible, including distance, intermediate, and near vision. Many types of premium IOL are now available, including accommodating IOL and multifocal IOLs. Multifocal IOLs can be categorized according to their design as diffractive lenses, refractive lenses, and lenses involving both diffractive and refractive designs [1, 2].

As a new multifocal IOL concept, refractive rotational asymmetry has been introduced into clinical practice for about a half decade, the main part of the lens behaves as a standard monofocal IOL, but in a specific sector of the lens, light is split into numerous foci. The Lentis Mplus LS-312 MF is the first commercially available refractive rotational asymmetry IOL and is a biconvex acrylic single-piece IOL containing a sector-shaped near-vision area with a +3.00 D addition (add) (+3 D for LS-312 MF 30; +1.5 D for LS-312 MF 15) [3] and an aspheric distance-vision zone. Since there is a smooth transition between the two zones, intermediate visual acuity might be improved to some extent [4, 5].

Several studies have compared the Lentis Mplus LS-312 MF30, LS-312 MF15, or LS-313 MF30 with other types of IOL [6–14]; however, the results have not always been consistent. To the best of our knowledge, this report represents the first meta-analysis of the clinical performance of refractive rotational asymmetry lenses.

## 2. Material and Methods

This study was registered at International Prospective Register of Systematic Reviews and was reported in accordance with the Preferred Reporting Items for Systematic Reviews and Meta-analysis (PRISMA).

### 2.1. Search Strategy and Screening Process

Two reviewers (Z. X. and W. L.) independently searched PubMed, EMBASE, the Cochrane Controlled Trials Register and Web of Science using the following search terms as keywords: segmental refractive multifocal intraocular lens, rotationally asymmetric multifocal intraocular lens, LENTIS Mplus, and SBL-3. No limits were put on the language of the publication. The full articles were carefully analysed after a preliminary review of the titles and abstracts. A third reviewer (Xu Chen) was asked to adjudicate when disagreement existed between Z. X. and W. L.

### 2.2. Eligibility

We included all studies comparing rotationally asymmetric multifocal IOLs and other IOLs used in patients undergoing cataract surgery and/or refractive lens exchange surgery. However, studies involving patients with coexisting pathology and previous IOL implantation were excluded.

### 2.3. Data Collection

Using a standardized data-collection form, two reviewers (X. X. and W. L.) independently extracted the data characteristics of the included studies; we attempted to obtain missing data by emailing the authors directly.

### 2.4. Quality Assessment

For all included cohort studies, the Newcastle-Ottawa Scale (NOS) [15] was used for quality assessment. The maximum NOS score is nine points, and a score of seven points indicates good quality. This scale includes three areas: patient selection (four points maximum), outcome assessment (two points maximum), and comparability (two points maximum).

### 2.5. Outcome Measures

Uncorrected distance visual acuity (UDVA), corrected distance visual acuity (CDVA), uncorrected intermediate visual acuity (UIVA), uncorrected near visual acuity (UNVA), distance-corrected near visual acuity (DCNVA), and corrected near visual acuity (CNVA) were recorded at logMAR; distance visual acuity was recorded at 4 or 6 m, intermediate visual acuity was recorded at 70, 63, or 66 cm, and near visual acuity was recorded at 40 or 33 cm. Distance, intermediate, and near visual acuity could also be determined based on defocus curves. Contrast sensitivity testing was performed under photopic conditions (85 cd/m^2^) and mesopic conditions (3 cd/m^2^). Data regarding MTF, Strehl ratio, higher-order aberrations (HOAs), and residual sphere and cylinder were also collected if provided. The MTF cut-off point represented the point where the spatial frequency was maximal and the Strehl ratio is the ratio of peak focal intensities in the aberrated and ideal ocular point spread function (PSF), both of which had a theoretic relationship with the visual quality [7].

### 2.6. Statistical Analysis

The data were analysed using Rev Manager Software (version 5.3; Cochrane Collaboration, Oxford, United Kingdom). Forest plots were used to present the results, and chi-square and *I* ^2^ tests were used to test for statistical heterogeneity; a random-effects meta-analysis was used when *I* ^2^ > 50%, and a fixed-effects models was used otherwise [16]. The weighted mean difference (WMD) with 95% (confidence intervals) CIs was calculated. Statistical significance was defined as a *P*-value of less than 0.05. And for visual acuity, 0.1 logMAR was to be assumed clinically significant [17].

## 3. Results

**Figure 1:**
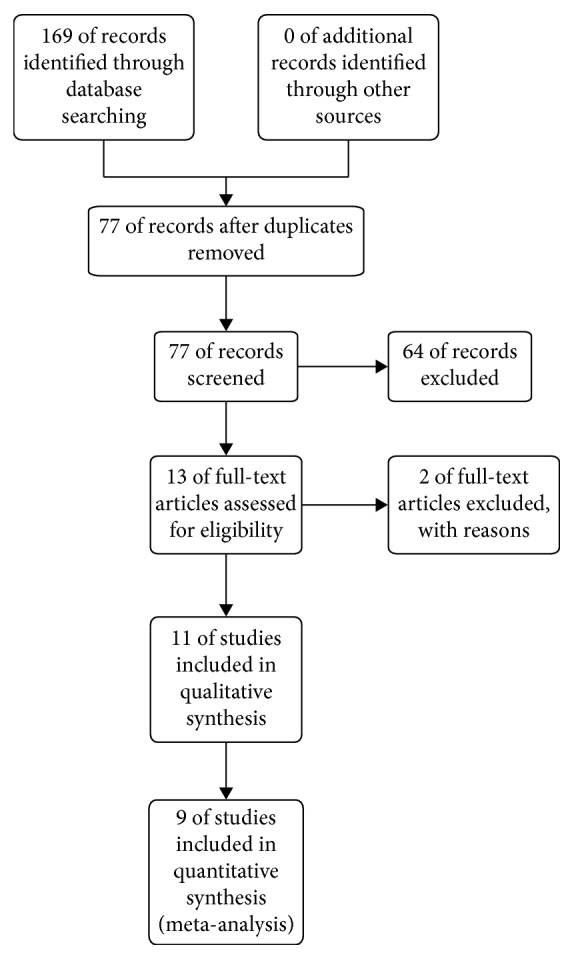
Study selection process of nonrandomized cohort trials.

Figure 1 shows a flow diagram of the included and excluded studies. The search strategy generated 169 potentially relevant studies, of which nine [6–14] were included in our quantitative synthesis; all nine studies were nonrandomized cohort studies.

### 3.1. Characteristics of the Included Studies

Table 1 shows the characteristics of the nine studies that met all inclusion criteria. All studies were comparative cohort trials and were performed in Europe, and all patients underwent cataract surgery except that in one study [14], some patients underwent refractive lens exchange surgery. Mplus IOLs (312 MF30, 313 MF30, and 312 MF30) were used in the refractive rotationally asymmetric multifocal IOL group, and spherical monofocal (Acri.Smart 48S) IOLs, accommodating IOLs (Crystalens HD), and refractive-diffractive bifocal IOLs (Acri.Lisa 366 and ReSTOR SN6AD 1/3) were used in comparison groups.

All but one of the nine studies had no missing cases [8], and all reported all of their main results; thus, eight studies [6, 7, 9–14] had three points for outcome assessment (three points maximum), and one study had two points [8]. All of the studies scored two points for comparability (two points maximum). One study had flaws in patient selection (four points maximum) [7] and did not match preoperative distance visual acuity and higher-order aberrations; thus, the study was scored as two points for patient selection. All other studies were scored as four points.

### 3.2. Quality of the Methodology Used

Table 2 shows the summary of outcomes (including the overall quality of evidence as assessed from GRADE/GDT).

### 3.3. Primary Outcome

The primary outcomes were distance visual acuity (UDVA), corrected distance visual acuity (CDVA), uncorrected intermediate visual acuity (UIVA), uncorrected near visual acuity (UNVA), distance-corrected near visual acuity (DCNVA) and corrected near visual acuity (CNVA), higher-order aberrations (HOAs), MTF cut-off, and Strehl ratio.

#### 3.3.1. Uncorrected Distance Visual Acuity (UDVA)

**Figure 2:**
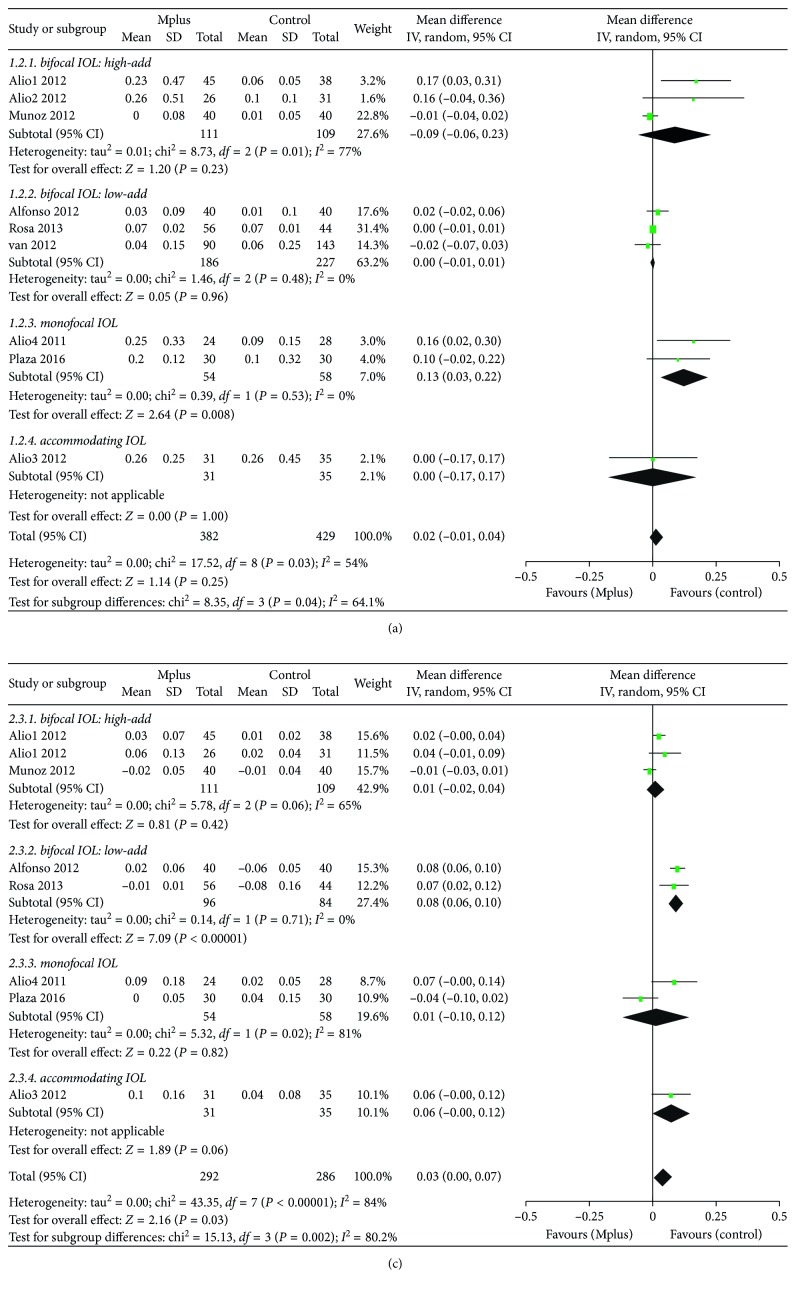
Meta-analysis of postoperative binocular uncorrected distance visual acuity (UDVA) (a), and corrected distance visual acuity (CDVA) (b). SD = standard deviation; CI = confidence interval.

Nine studies [6–14] reported UDVA. The mean UDVA in the Mplus group was 0.120 ± 0.269, which was not significantly different from that in the control group (WMD: 0.02, 95% CI: −0.01 to 0.04, *P*=0.25) (Figure 2). The quality of the evidence was high (Figure S1). Subgroup analysis according to the type of IOL employed in the control group was conducted. Mplus provided significantly worse UDVA than spherical monofocal IOLs (WMD: 0.13, 95% CI: 0.03 to 0.22, *P*=0.008). The quality of the evidence is shown in Figure S1.

#### 3.3.2. Corrected Distance Visual Acuity (CDVA)

Eight studies [6–13] reported CDVA. The mean CDVA in the Mplus group was significantly worse than that in the control group (WMD: 0.03, 95% CI: 0.00 to 0.07, *P*=0.03) (Figure 2); however, the difference was not clinically significant. Subgroup analysis according to the type of IOL employed in the control group was also conducted. Mplus resulted in significantly worse CDVA than low-add refractive-diffractive bifocal IOLs (WMD: 0.08, 95% CI: 0.06 to 0.10, *P* < 0.00001). The quality of the evidence is shown in Figure S2.

#### 3.3.3. Uncorrected Intermediate Visual Acuity (UIVA)

**Figure 3:**
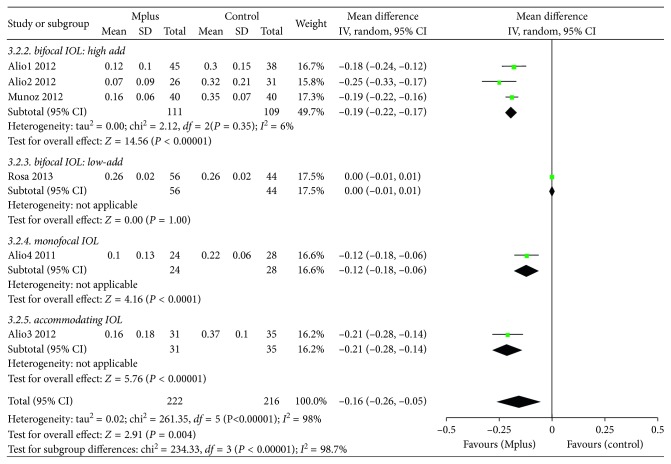
Meta-analysis of postoperative binocular uncorrected intermediate visual acuity (UIVA).

Eight studies [6–13] reported UIVA. The mean UIVA in the Mplus group was 0.160 ± 0.118, which was significantly better than that in the control group (WMD: −0.16, 95% CI: −0.26 to −0.05, *P*=0.004) (Figure 3). Subgroup analysis according to the type of IOL employed in the control group was also conducted. Mplus resulted in significantly better UIVA than high-add refractive-diffractive bifocal IOLs (WMD: −0.19, 95% CI: −0.22 to −0.17, *P* < 0.00001), spherical monofocal IOLs (WMD: −0.12, 95% CI: −0.18 to −0.06, *P* < 0.0001), and accommodating IOLs (WMD: −0.21, 95% CI: −0.28 to −0.14, *P* < 0.00001). The quality of the evidence is shown in Figure S3.

#### 3.3.4. Uncorrected near Visual Acuity **(**UNVA**)**

**Figure 4:**
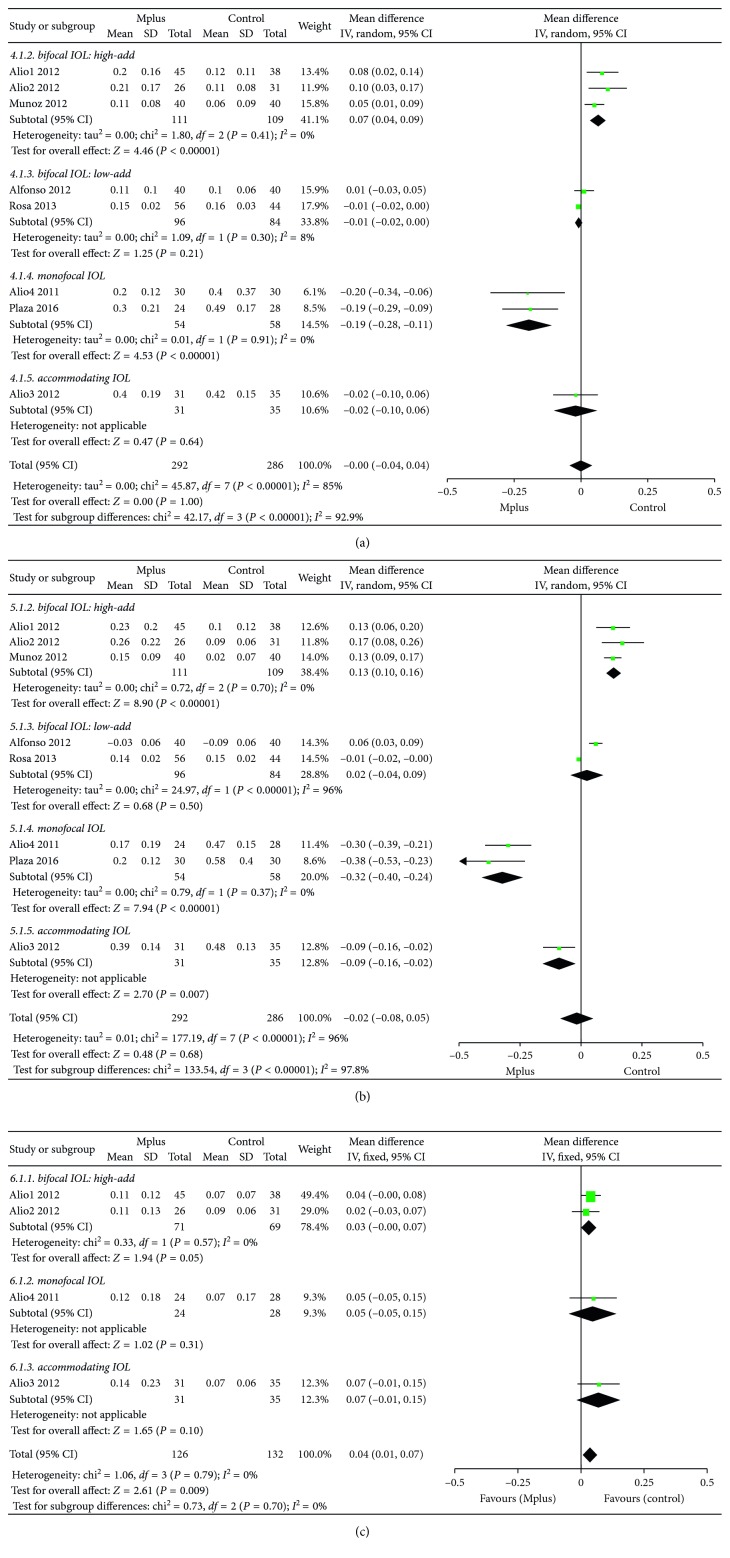
Meta-analysis of postoperative binocular uncorrected distance visual acuity (UDVA) (a), distance-corrected near visual acuity (DCNVA) (b), and corrected near visual acuity (CNVA) (c). SD = standard deviation; CI = confidence interval.

Eight studies [6–13] reported UNVA. The mean UNVA in the Mplus group was 0.196 ± 0.158, almost the same as that in the control group (WMD: −0.00, 95% CI: −0.04 to 0.04, *P*=1) (Figure 4). Subgroup analysis according to the type of IOL used in the control group was also conducted. Mplus provided significantly worse UNVA than high-add refractive-diffractive bifocal IOLs (WMD: 0.07, 95% CI: 0.04 to 0.09, *P* < 0.00001), although the difference was not very clinically significant. However, Mplus provided significantly better UNVA than spherical monofocal IOLs (WMD: −0.19, 95% CI: −0.28 to −0.11, *P* < 0.00001). The quality of the evidence is shown in Figure S4.

#### 3.3.5. Distance-Corrected Near Visual Acuity (DCNVA)

Eight studies [6–13] reported DCNVA. The mean DCNVA in the Mplus group was almost the same as that in the control group (WMD: −0.02, 95% CI: −0.08 to 0.05, *P*=0.63) (Figure 4). Subgroup analysis according to the type of IOL used in the control group was also conducted. Mplus resulted in significantly worse DCNVA than high-add refractive-diffractive bifocal IOLs (WMD: 0.13, 95% CI: 0.10 to 0.16, *P* < 0.00001), and the difference was clinically significant. However, Mplus resulted in significantly better DCNVA than spherical monofocal IOLs (WMD: −0.32, 95% CI: −0.40 to −0.24, *P* < 0.00001). The quality of the evidence is shown in Figure S5.

#### 3.3.6. Corrected Near Visual Acuity (CNVA)

Four studies [7, 8, 11, 12] reported CNVA. The mean CNVA in the Mplus group was worse than that in the control group (WMD: 0.04, 95% CI: 0.01 to 0.07, *P*=0.009) (Figure 4), although the difference was not clinically significant. Subgroup analysis according to the type of IOL used in the control group was also conducted. Mplus resulted in significantly worse CNVA than high-add refractive-diffractive bifocal IOLs (WMD: 0.03, 95% CI: −0.00 to 0.07, *P*=0.05), although the difference was not clinically significant. The quality of the evidence is shown in Figure S6.

#### 3.3.7. Higher-Order Aberrations (HOAs)

**Figure 5:**
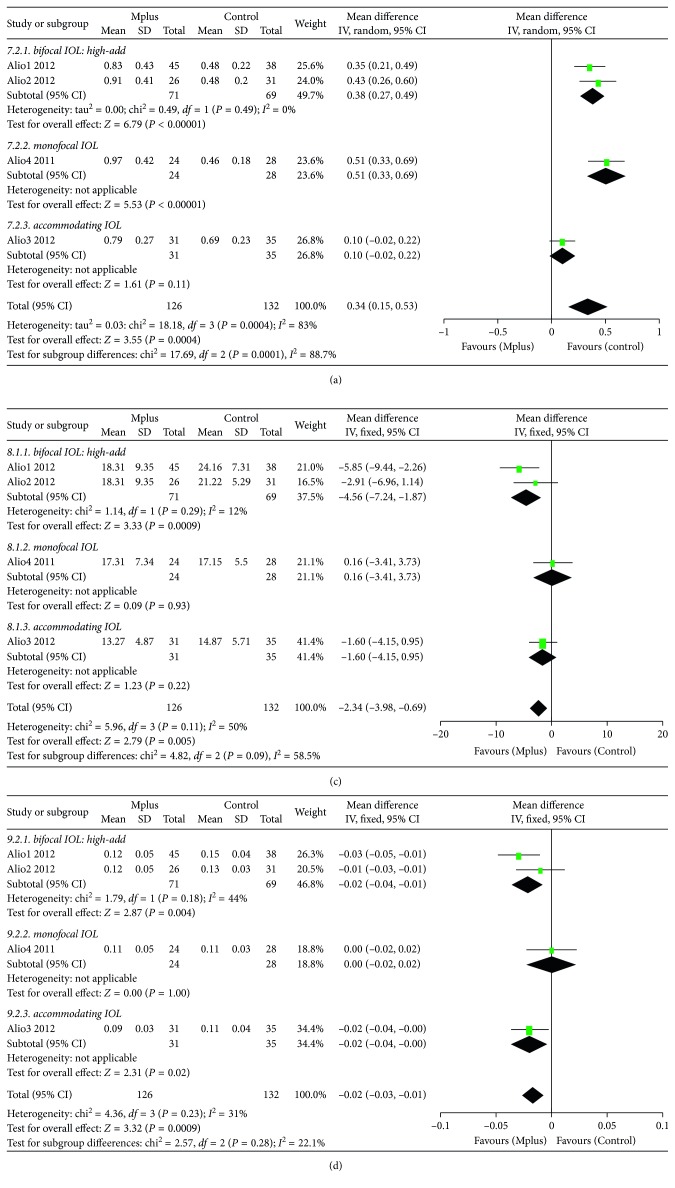
Meta-analysis of postoperative binocular uncorrected distance visual acuity (UDVA) (a), distance-corrected near visual acuity (DCNVA) (b), and corrected near visual acuity (CNVA) (c). SD = standard deviation; CI = confidence interval.

Four studies [7, 8, 11, 12] reported residual higher-order aberrations (HOAs). The HOAs of the fours studies were recorded by the same ocular aberrometry (COAS; Wavefront Sciences Inc, Albuquerque, New Mexico). The mean number of HOAs in the Mplus group was significantly higher than that in the control group (WMD: 0.34, 95% CI: 0.15 to 0.53, *P*=0.0004) (Figure 5). Subgroup analysis according to the type of IOL used in the control group was also conducted. Mplus resulted in significantly higher HOAs than high-add refractive-diffractive bifocal IOLs (WMD: 0.38, 95% CI: 0.27 to 0.49, *P* < 0.00001) and spherical monofocal IOLs (WMD: 0.51, 95% CI: 0.33 to 0.69, *P*=0.0004). The quality of the evidence is shown in Figure S7.

#### 3.3.8. MTF Cut-Off

Higher values of MTF cut-off indicate better vision quality. Four studies [7, 8, 11, 12] reported residual MTF cut-off. The mean MTF cut-off in the Mplus group was significantly lower than that in the control group (WMD: −2.34, 95% CI: −3.98 to −0.69, *P*=0.005) (Figure 5). Subgroup analysis according to the type of IOL used in the control group was also conducted. Mplus resulted in a significantly lower MTF cut-off than high-add refractive-diffractive bifocal IOLs (WMD: −4.56, 95% CI: −7.24 to −1.87, *P*=0.0009). The quality of the evidence is shown in Figure S8.

#### 3.3.9. Strehl Ratio

Higher values of the Strehl ratio indicate better vision quality. Four studies [7, 8, 11, 12] reported the residual Strehl ratio. The mean Strehl ratio in the Mplus group was significantly lower than that in the control group (WMD: −0.02, 95% CI: −0.03 to −0.01, *P*=0.0009) (Figure 5). Subgroup analysis according to the type of IOL used in the control group was also conducted. Mplus resulted in significantly lower Strehl ratios than high-add refractive-diffractive bifocal IOLs (WMD: −0.02, 95% CI: −0.04 to −0.01, *P*=0.004) and accommodating IOLs (WMD: −0.02, 95% CI: −0.04 to −0.00, *P*=0.02). The quality of the evidence is shown in Figure S9.

#### 3.3.10. Defocus Curve

Seven studies [6–8, 11–14] reported defocus curves, and a summary of the results is shown in Table 3. The results of defocus curve were consistent with the visual acuity results.

#### 3.3.11. Contrast Sensitivity

Six studies [7–9, 11–13] reported contrast sensitivity, and a summary of the results is shown in Table 4. Under the photopic condition, high-add Mplus IOLs yielded significantly better performance at 12 and 18 c/d than high-add bifocal IOLs but significantly worse performance at 12 and 18 c/d than low-add bifocal IOLs. Low-add Mplus IOLs resulted in significantly worse performance at 3, 6, 12, and 18 c/d than accommodating IOLs. Under the low conditions, Mplus IOLs had a tendency to provide worse results than spherical monofocal IOLs and accommodating IOLs.

### 3.4. Secondary Outcomes

The secondary outcomes were residual sphere and cylinder.

#### 3.4.1. Residual Sphere

Eight studies [7–14] reported residual sphere. The mean residual sphere in the Mplus group was significantly lower than that in the control group (WMD: −0.12, 95% CI: −0.23 to −0.02, *P*=0.02). The difference, however, was not clinically significant.

#### 3.4.2. Residual Cylinder

Eight studies [7–14] reported residual cylinder. The mean residual sphere in the Mplus group was not significantly different from that in the control group (WMD: 0.18, 95% CI: −0.22 to 0.57, *P*=0.38).

## 4. Discussion

This is a meta-analysis which compares a refractive rotationally asymmetric multifocal intraocular lens (Mplus IOL) and an accommodative, a monofocal, or a bifocal IOL, respectively. Outcome parameters such as uncorrected distance visual acuity (UDVA), uncorrected intermediate visual acuity (UIVA), uncorrected near visual acuity (UNVA), etc., were determined. Our findings suggested that asymmetric multifocal IOLs provide good, but not perfect, results in terms of objective visual performance and vision quality.

Uncorrected distance visual acuity (UDVA) following implantation of Mplus IOLs was good and not significantly different from those following the implantation of accommodating IOLs and refractive-diffractive bifocal IOLs. In an extensive study including 9366 eyes by Venter et al. [18], the mean UDVA of Mplus IOLs was 0.054 ± 0.146 logMAR; the results were similar to those obtained in our study. However, the UDVA performance of Mplus IOLs was inferior to that of spherical monofocal IOLs in our study, and the difference between them was not only statistically (*P*=0.008) but also clinically (0.13 logMAR) significant. Thus, Mplus IOLs still have room to improve in terms of distance visual acuity.

Mplus IOLs exhibited better uncorrected intermediate visual acuity (UIVA) than spherical monofocal IOLs, accommodating IOLs, and high-add refractive-diffractive bifocal IOLs (+4 D, +3.75 D). The difference between Mplus IOLs and other IOLs (spherical monofocal, accommodating, and high-add refractive-diffractive bifocal IOLs) was clinically significant (0.12 logMAR, 0.21 logMAR, and 0.19 logMAR, respectively). It is worth noting that the refractive rotationally asymmetric multifocal IOL that was compared with the accommodating IOLs was a low-add Mplus IOL (+1.5 D), which exhibited superior intermediate visual performance than high-add Mplus IOLs (+3.0 D) [3], and this may have partially contributed to the clear advantage over accommodating IOLs. Further analysis may be needed to compare high-add Mplus IOLs (+3.0 D) and accommodating IOLs. In a study by Munoz et al. the mean UIVA of Mplus IOLs was 0.13 ± 0.12 logMAR [19], and these results were similar to those obtained in our study. Thus, satisfying intermediate visual acuity was achieved using Mplus IOLs. Two reasons might explain the improvement of UIVA: 1, the transition zone between distance- and near-vision sectors was smooth and gradual; 2, the slight induction of HOAs (mainly coma and trefoil) may provide a certain depth of focus.

Mplus IOLs exhibited good uncorrected near visual acuity (UNVA). Unsurprisingly, Mplus IOLs performed better than spherical monofocal IOLs. Furthermore, Mplus IOLs also performed better than accommodating IOLs, but the difference was not as clinically significant (0.09 logMAR). This was an encouraging result since a previous meta-analysis had already shown that accommodating IOLs can restore satisfying near vision without compromising distance vision [20]. However, almost no difference was found between Mplus and low-add refractive-diffractive bifocal IOLs (+3 D). Finally, Mplus had a statistically (*P* < 0.00001) inferior performance to that of high-add refractive-diffractive bifocal IOLs (+4 D, +3.75 D), but this difference was not clinically significant (0.07 logMAR). In the extensive study by Venter et al. [18], the mean UNVA of Mplus IOLs was 0.213 ± 0.173 logMAR, and the results were similar in our study. Thus, both refractive-diffractive bifocal IOLs [2, 21, 22] and Mplus provide excellent near visual performance; in addition, accommodating IOLs also performed well, and all were significantly better than spherical monofocal IOLs.

Higher-order aberrations were significantly greater in eyes that were implanted with Mplus IOLs than in those implanted with refractive-diffractive bifocal IOLs and spherical monofocal IOLs, which exhibit strong spherical aberrations [23, 24]. Aberrations were tend to be significantly greater in eyes that had been implanted with Mplus IOLs than in those that had been implanted with accommodating IOLs. The presence of strong intraocular higher-order aberrations in Mplus IOLs is usually attributed to coma and trefoil [4, 25], which in turn are usually attributed to its design, which involves vertical asymmetric optical geometry [4, 26]. IOL tilt is caused by ineffectiveness in stabilizing the lens [12, 27] or by placing the near segment inferiorly with slight nasal deviation as recommended by manufacturers' guidelines [25, 28, 29].

IOLs with a rotationally asymmetrical design contributes significantly towards vision quality [30]; however, this lens type exhibited decreased vision quality compared with accommodating IOLs and refractive-diffractive bifocal IOLs. Moreover, no significant differences were found in MTF cut-off and Strehl ratio between Mplus and the spherical monofocal IOLs, whose vision quality was limited by residual spherical aberrations. The large amount of residual higher-order aberrations may be the main reason for the limited vision quality provided by Mplus IOLs, as mentioned above.

Contrast sensitivity is a well-recognized parameter that is used to assess the quality of vision of pseudophakic eyes and reflects the lowest contrast level that can be detected for a given size target [31]. Reduced contrast sensitivity is one of the main reasons for dissatisfaction in postoperative cataract patients [32]. Rotationally asymmetrical designs aim to increase contrast sensitivity and alleviate photopic phenomena [33], and this goal is achieved to some extent; however, lenses of this type still face decreased contrast sensitivity. The increased higher-order aberrations mentioned above may partially attribute to the decreased contrast sensitivity of Mplus IOLs.

This study has several limitations. First, further analysis is required regarding many other related types of IOLs, such as Mplus (+2 D or +1.5 D) and SBL-3 IOLs in the refractive rotationally asymmetric multifocal groups, trifocal IOLs and +2.5 D bifocal IOLs in the multifocal groups, and 1CU IOLs and wiol-cf IOLs in the accommodating IOLs group. Second, one study [14] received grants from Alcon Laboratories (Fort Worth, TX, USA). Third, publication bias may occur, but we failed to do funnel plots because the number of the studies included in each subgroup is limited. Fourth, four referred articles reporting higher-order aberrations, MTF cut-off, and Strehl ratio included in the present meta-analysis have been published by the same research group.

To conclude, the refractive rotationally asymmetric multifocal IOLs provided improved intermediate visual acuity and satisfying distance visual acuity, as well as acceptable near visual acuity, all of which led to less need for spectacles. High-add Mplus IOLs provided superior intermediate and near but inferior distance visual performance compared to spherical monofocal IOLs and provided superior intermediate but inferior near visual performance compared to high-add refractive-diffractive bifocal IOLs. Low-add Mplus IOLs provided superior intermediate and near visual performance compared to accommodating IOLs. However, Mplus IOLs resulted in some residual higher-order aberrations that might affect the corrected visual acuity and quality of vision. Thus, we recommend that asymmetric multifocal IOLs be considered an important member of the IOL family.

## Figures and Tables

**Table 1:** Characteristics of the studies (*n* = 9) included in the meta-analysis.

Study	Site	Design	Procedure	Included eyes: experimental group/control group	IOL in the experimental group	IOL in the control group	Newcastle-Ottawa scale scores	Follow-up (months)
Munoz et al. [6]	Spain	C	Cataract	40/40	Mplus LS-312 MF30	Acri.Lisa 366	Patient selection: 4 Comparability: 2 Outcome assessment: 3	6

Alio et al. [7]	Spain	C	Cataract	45/38	Mplus LS-312 MF30	Acri.Lisa 366	Patient selection: 2 Comparability: 2 Outcome assessment: 3	3

Alio et al. [8]	Spain	C	Cataract	26/31	Mplus LS-312 MF30	ReSTOR SN6AD3	Patient selection: 4 Comparability: 2 Outcome assessment: 2	3

Rosa et al. [9]	Portugal	C	Cataract	56/44	Mplus LS-312 MF30	ResSTOR SN6AD1	Patient selection: 4 Comparability:2 Outcome assessment: 3	3

Plaza et al. [10]	Spain	C	Cataract	30/30	Mplus LS-313 MF30	Acri.Smart 48S	Patient selection: 4 Comparability: 2 Outcome assessment: 3	3

Alio et al. [11]	Spain	C	Cataract	31/35	Mplus LS-312 MF15	Crystalens HD	Patient selection: 4 Comparability: 2 Outcome assessment: 3	3

Alio et al. [3, 12]	Spain	C	Cataract	24/28	Mplus LS-312 MF30	Acri.Smart 48S	Patient selection: 4 Comparability: 2 Outcome assessment: 3	3

Alfonso et al. [13]	Spain	C	Cataract	40/40	Mplus LS-312 MF30	ResSTOR SN6AD1	Patient selection: 4 Comparability: 2 Outcome assessment: 3	6

van der Linden et al. [14]	The Netherlands	C	RLE/cataract	90/143	Mplus LS-312 MF30	ResSTOR SN6AD1	Patient selection: 4 Comparability: 2 Outcome assessment: 3	3

C = comparative cohort trials; RLE = refractive lens change.

**Table 2:** Summary of the main outcomes included in the meta-analysis.

Outcome	Risk for Mplus	Number of participants (studies)	Importance	Quality	Comments
UDVA	The intervention group was 0.02 higher (0.01 lower to 0.04 higher)	811 (9 studies)	CRITICAL	⊕ ⊕ ⊕ ⊕ high	See subgroup analysis in Figure 2(a)
CDVA	The intervention group was 0.03 higher (0 to 0.07 higher)	578 (8 studies)	CRITICAL	⊕ ⊕ ⊕ ⊝ moderate	See subgroup analysis in Figure 2(b)
UIVA	The intervention group was 0.16 lower (0.26 to 0.05 lower)	438 (6 studies)	CRITICAL	⊕ ⊕ ⊕ ⊕ high	See subgroup analysis in Figure 3
UNVA	The intervention group was 0 higher (0.04 lower to 0.04 higher)	578 (8 studies)	CRITICAL	⊕ ⊕ ⊕ ⊝ moderate	See subgroup analysis in Figure 4(a)
DCNVA	The intervention group was 0.02 lower (0.08 lower to 0.05 higher)	578 (8 studies)	CRITICAL	⊕ ⊕ ⊕ ⊝ moderate	See subgroup analysis in Figure 4(b)
CNVA	The intervention group was 0.04 higher (0.01 to 0.07 higher)	258 (4 studies)	CRITICAL	⊕ ⊕ ⊕ ⊝ ⊝ low	See subgroup analysis in Figure 4
HOA	The intervention group was 0.34 higher (0.15 to 0.53 higher)	258 (4 studies)	IMPORTANT	⊕ ⊕ ⊕ ⊕ high	See subgroup analysis in Figure 5(a)
MTF cut-off	The intervention group was 2.46 lower (4.84 to 0.07 lower)	258 (4 studies)	IMPORTANT	⊕ ⊕ ⊕ ⊝ moderate	See subgroup analysis in Figure 5(b)
Strehl ratio	The intervention group was 0.4 standard deviations lower (0.65 to 0.15 lower)	258 (4 studies)	IMPORTANT	⊕ ⊕ ⊕ ⊝ moderate	See subgroup analysis in Figure 5

UDVA = uncorrected distance visual acuity; UIVA = uncorrected intermediate visual acuity; UNVA = uncorrected near visual acuity.

**Table 3:** Comparison of defocus curves between the Mplus group and the control group.

Study	Mplus group	Control group	Mplus IOLs provided better performance	Control group provided better performance
Munoz et al. [6]	Mplus LS-312 MF30	High-add bifocal IOL: Acri.Lisa 366	In 3, 2.5, 2, 1.5, 1, 0.5, 0, −0.5^*∗*^, −1^*∗*^, −1.5^*∗*^, −2^*∗*^, −2.5^*∗*^ D	In −3, −3.5, −4, −4.5, −5 D

Alio et al. [7]	Mplus LS-312 MF30	High-add bifocal IOL: Acri.Lisa 366	In 1.5^*∗*^, 1, −1^*∗*^, −1.5^*∗*^, −2^*∗*^, −3, −3.5, −4, −4.5 D	In 0.5, 0, −0.5, −2.5 D

Alio et al. [8]	Mplus LS-312 MF30	High-add bifocal IOL: ReSTOR SN6AD3	In 1.5, −1^*∗*^, −1.5^*∗*^, −2^*∗*^, −2.5^*∗*^, −3^*∗*^, −3.5^*∗*^ D	In 0.5, 1, −0.5, −4, −4.5 D

Alfonso et al. [13]	Mplus LS-312 MF30	Low-add bifocal IOL: ReSTOR SN6AD1	In 2, 1.5, 1, −1, −1.5, D	In 0.5, 0^*∗*^, −0.5^*∗*^, −2^*∗*^, −2.5^*∗*^, 3^*∗*^, −3.5^*∗*^, −4^*∗*^ D

van der Linden et al. [14]	Mplus LS-312 MF30	Low-add bifocal IOL: ReSTOR SN6AD1	In 0, −1.5 D	In −2, −2.5^*∗*^, −3^*∗*^ D

Alio et al. [3, 12]	Mplus LS-312 MF30	Spherical monofocal IOL: Acri.Smart 48S	In 2.5^*∗*^, 2^*∗*^, 1.5^*∗*^, 1^*∗*^, 0.5^*∗*^, 0^*∗*^, −0.5^*∗*^, −1^*∗*^, −1.5^*∗*^, −3.5 D	In −2^*∗*^, −2.5^*∗*^, −3^*∗*^, −4, −4.5 D

Alio et al. [11]	Mplus LS-312 MF15	Accommodating IOL: Crystalens HD	In 1.5, 1, 0.5, 0, −0.5, −1^*∗*^, −1.5^*∗*^, −2^*∗*^, −2.5^*∗*^, −3^*∗*^ D	—

^*∗*^Significantly different.

**Table 4:** Comparison of contrast sensitivity between the Mplus group and the control group under photopic and low conditions.

Conditions	Study	Mplus group	Control group	Mplus IOLs provided better performance	Control group provided better performance
Photopic conditions	Alio et al. [7]	Mplus LS-312 MF30	High-add bifocal IOL: Acri.Lisa 366	In 3, 6, 12^*∗*^, 18^*∗*^ c/d	—

Photopic conditions	Alio et al. [8]	Mplus LS-312 MF30	High-add bifocal IOL: ReSTOR SN6AD3	In 3, 6^*∗*^, 12^*∗*^, 18^*∗*^ c/d	—

Photopic conditions	Alfonso et al. [13]	Mplus LS-312 MF30	Low-add bifocal IOL: ReSTOR SN6AD1	In 6 c/d	In 3, 12^*∗*^, 18^*∗*^ c/d

Photopic conditions	Rosa et al. [9]	Mplus LS-312 MF30	Spherical monofocal IOL: Acri.Smart 48S	—	In 0.6, 1.1, 2.2^*∗*^, 3.4^*∗*^, 7.1^*∗*^, 23.6 c/d

Photopic conditions	Alio et al. [3, 12]	Mplus LS-312 MF30	Spherical monofocal IOL: Acri.Smart 48S	In 3, 6 c/d	In 12, 18 c/d

Photopic conditions	Alio et al. [11]	Mplus LS-312 MF15	Accommodating IOL: Crystalens HD	—	In 3^*∗*^, 6^*∗*^, 12^*∗*^, 18^*∗*^ c/d

Low conditions	Alio et al. [7]	Mplus LS-312 MF30	High-add bifocal IOL: Acri.Lisa 366	In 3 c/d	In 6, 12, 18 c/d

Low conditions	Alio et al. [8]	Mplus LS-312 MF30	High-add bifocal IOL: ReSTOR SN6AD3	In 3, 6, 12, 18 c/d	—

Low conditions	Alfonso et al. [13]	Mplus LS-312 MF30	Low-add bifocal IOL: ReSTOR SN6AD1	In 3, 6, 12 c/d	In 18 c/d

Low conditions	Rosa et al. [9]	Mplus LS-312 MF30	Spherical monofocal IOL: Acri.Smart 48S	—	In 0.6, 1.1, 2.2^*∗*^, 3.4, 7.1, 23.6 c/d

Low conditions	Alio et al. [3, 12]	Mplus LS-312 MF30	Spherical monofocal IOL: Acri.Smart 48S	—	In 3, 6, 12, 18 c/d

Low conditions	Alio et al. [11]	Mplus LS-312 MF15	Accommodating IOL: Crystalens HD	In 18 c/d	In 3, 6, 12 c/d

^*∗*^Significantly different.

## Supplementary Materials

Supplementary Materials Figure S1: quality of evidence (UDVA). Figure S2: quality of evidence (CDVA). Figure S3: quality of evidence (UIVA). Figure S4: quality of evidence (UNVA). Figure S5: quality of evidence (DCNVA). Figure S6: quality of evidence (CNVA). Figure S7: quality of evidence, Higher-order aberrations (HOAs). Figure S8: quality of evidence (MTF cut-off). Figure S9: quality of evidence (Strehl ratio).
