# Supplementary figures and images for: Comparison of the Clinical Performance of Refractive Rotationally Asymmetric Multifocal IOLs with Other Types of IOLs: A Meta-Analysis
Source: J Ophthalmol. 2018 Sep 27;2018:4728258. doi: 10.1155/2018/4728258 (PMC6181000; doi:10.1155/2018/4728258)

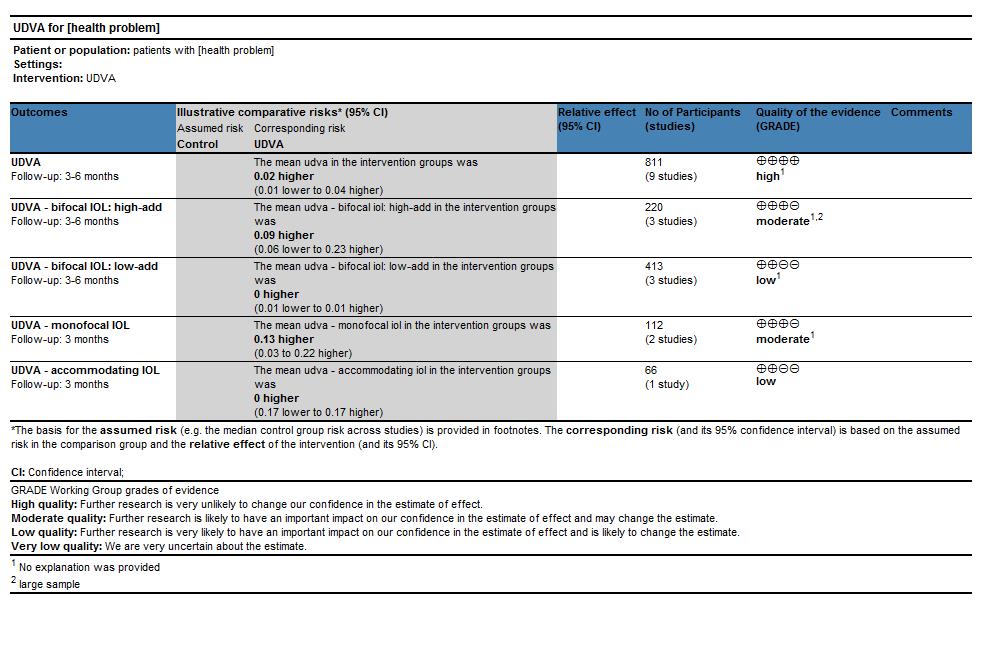

Supplement: Supplementary Materials — Figure S1: quality of evidence (UDVA). Figure S2: quality of evidence (CDVA). Figure S3: quality of evidence (UIVA). Figure S4: quality of evidence (UNVA). Figure S5: quality of evidence (DCNVA). Figure S6: quality of evidence (CNVA). Figure S7: quality of evidence, Higher-order aberrations (HOAs). Figure S8: quality of evidence (MTF cut-off). Figure S9: quality of evidence (Strehl ratio). [file 4728258.f1.zip › 4728258.f1/S1.jpeg]

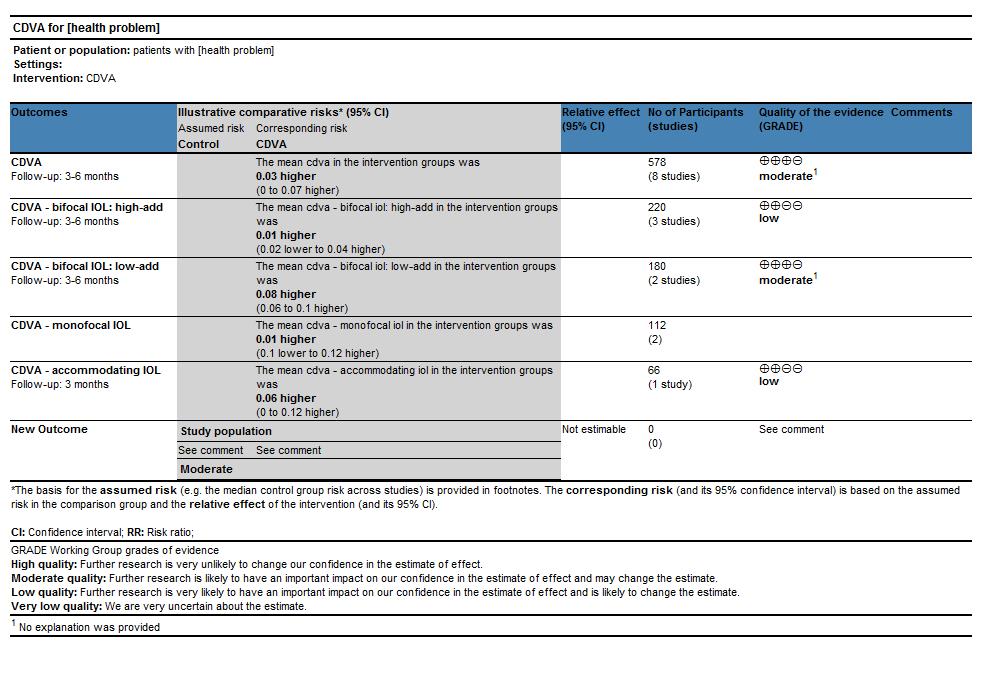

Supplement: Supplementary Materials — Figure S1: quality of evidence (UDVA). Figure S2: quality of evidence (CDVA). Figure S3: quality of evidence (UIVA). Figure S4: quality of evidence (UNVA). Figure S5: quality of evidence (DCNVA). Figure S6: quality of evidence (CNVA). Figure S7: quality of evidence, Higher-order aberrations (HOAs). Figure S8: quality of evidence (MTF cut-off). Figure S9: quality of evidence (Strehl ratio). [file 4728258.f1.zip › 4728258.f1/s2.jpeg]

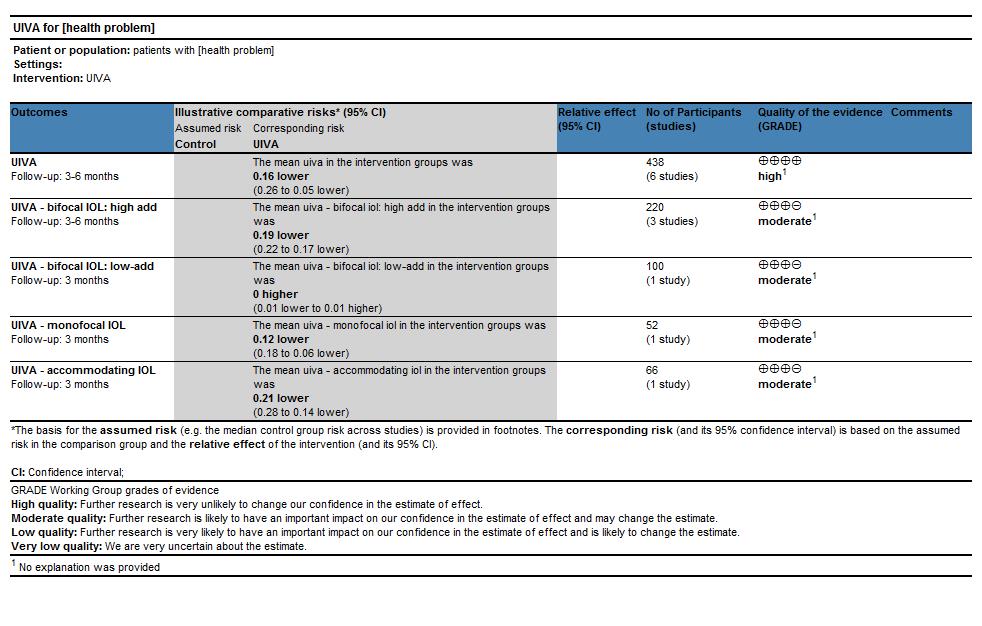

Supplement: Supplementary Materials — Figure S1: quality of evidence (UDVA). Figure S2: quality of evidence (CDVA). Figure S3: quality of evidence (UIVA). Figure S4: quality of evidence (UNVA). Figure S5: quality of evidence (DCNVA). Figure S6: quality of evidence (CNVA). Figure S7: quality of evidence, Higher-order aberrations (HOAs). Figure S8: quality of evidence (MTF cut-off). Figure S9: quality of evidence (Strehl ratio). [file 4728258.f1.zip › 4728258.f1/s3.jpeg]

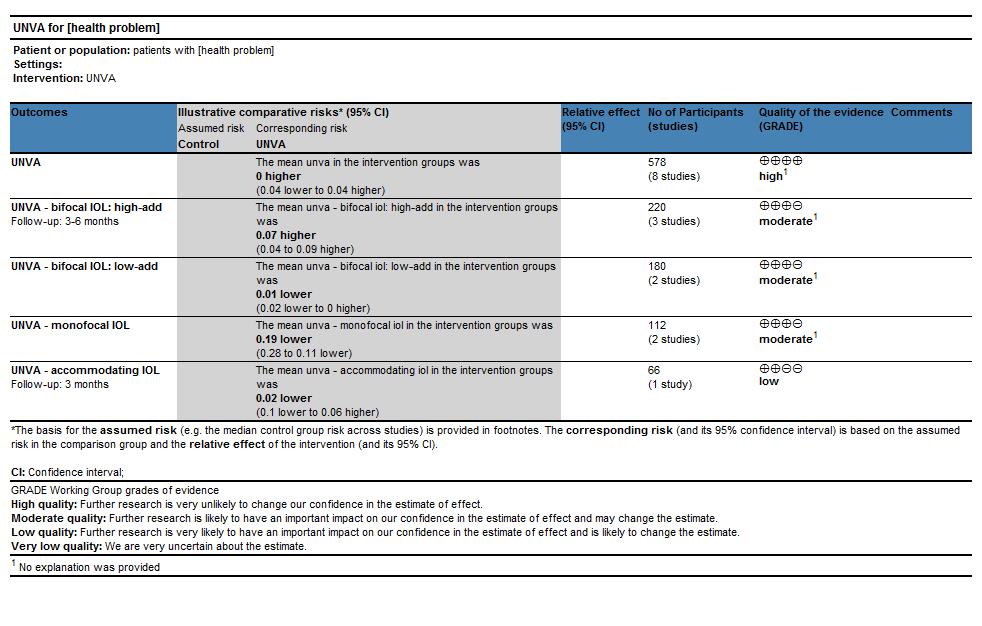

Supplement: Supplementary Materials — Figure S1: quality of evidence (UDVA). Figure S2: quality of evidence (CDVA). Figure S3: quality of evidence (UIVA). Figure S4: quality of evidence (UNVA). Figure S5: quality of evidence (DCNVA). Figure S6: quality of evidence (CNVA). Figure S7: quality of evidence, Higher-order aberrations (HOAs). Figure S8: quality of evidence (MTF cut-off). Figure S9: quality of evidence (Strehl ratio). [file 4728258.f1.zip › 4728258.f1/s4.jpeg]

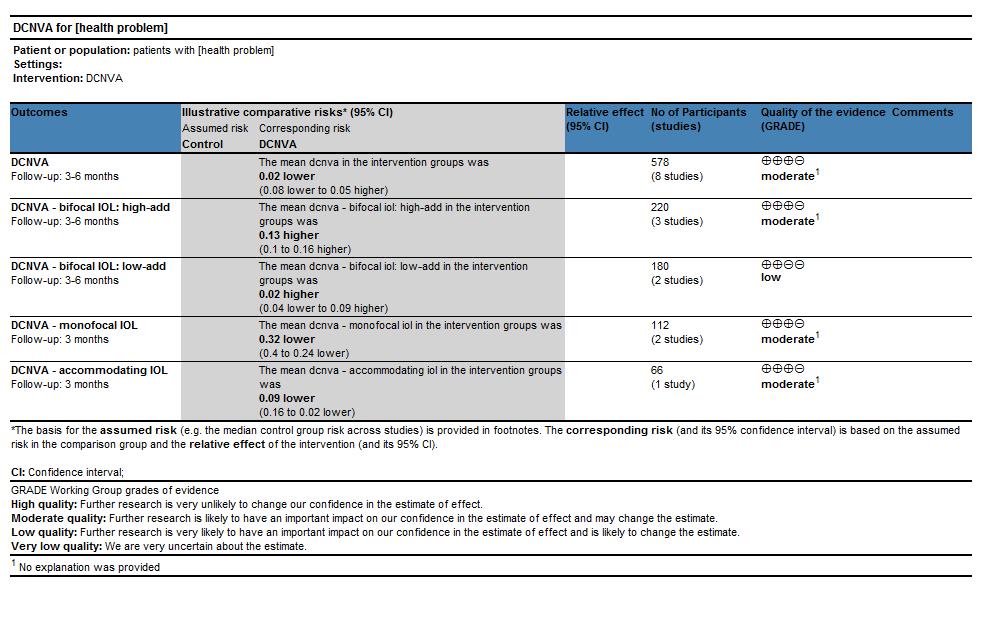

Supplement: Supplementary Materials — Figure S1: quality of evidence (UDVA). Figure S2: quality of evidence (CDVA). Figure S3: quality of evidence (UIVA). Figure S4: quality of evidence (UNVA). Figure S5: quality of evidence (DCNVA). Figure S6: quality of evidence (CNVA). Figure S7: quality of evidence, Higher-order aberrations (HOAs). Figure S8: quality of evidence (MTF cut-off). Figure S9: quality of evidence (Strehl ratio). [file 4728258.f1.zip › 4728258.f1/s5.jpeg]

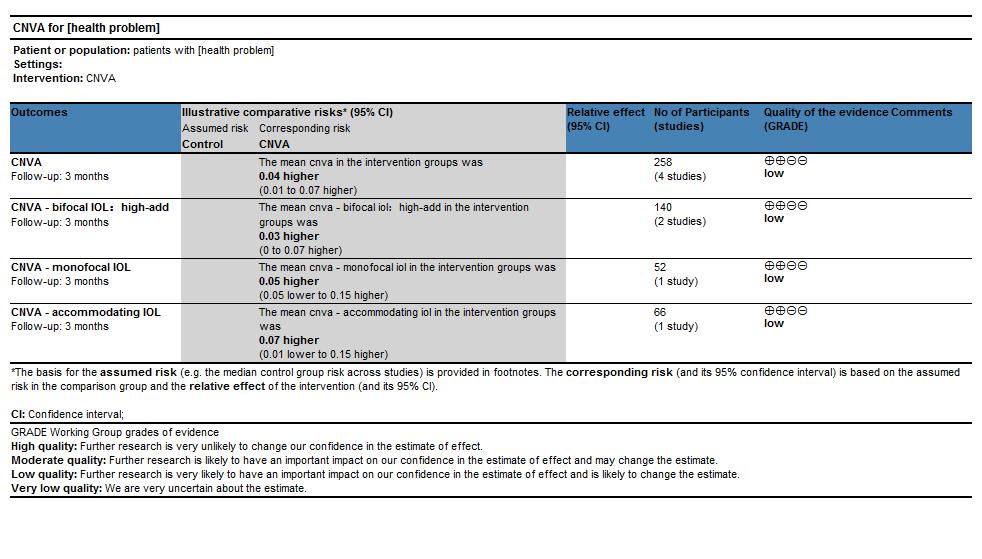

Supplement: Supplementary Materials — Figure S1: quality of evidence (UDVA). Figure S2: quality of evidence (CDVA). Figure S3: quality of evidence (UIVA). Figure S4: quality of evidence (UNVA). Figure S5: quality of evidence (DCNVA). Figure S6: quality of evidence (CNVA). Figure S7: quality of evidence, Higher-order aberrations (HOAs). Figure S8: quality of evidence (MTF cut-off). Figure S9: quality of evidence (Strehl ratio). [file 4728258.f1.zip › 4728258.f1/s6.jpeg]

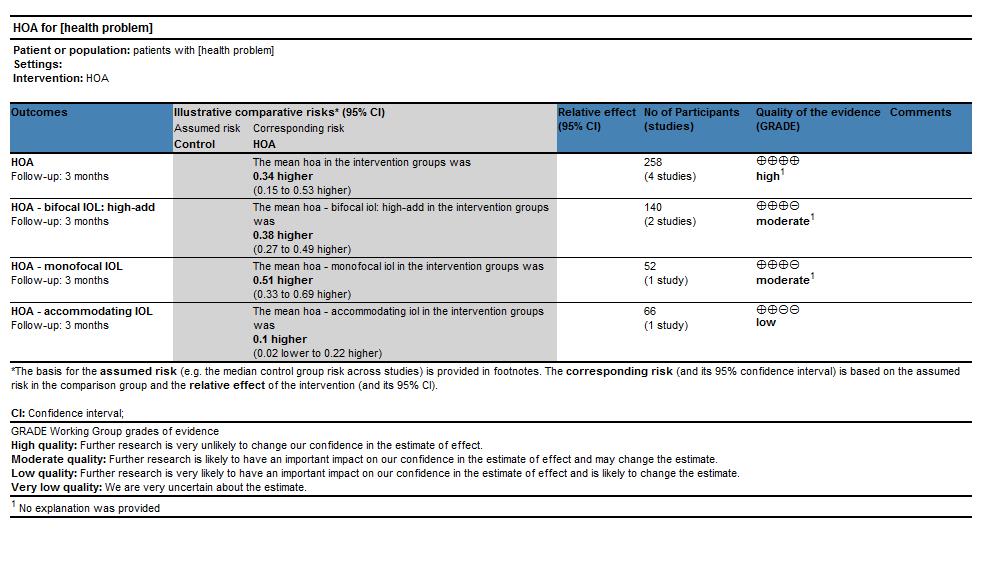

Supplement: Supplementary Materials — Figure S1: quality of evidence (UDVA). Figure S2: quality of evidence (CDVA). Figure S3: quality of evidence (UIVA). Figure S4: quality of evidence (UNVA). Figure S5: quality of evidence (DCNVA). Figure S6: quality of evidence (CNVA). Figure S7: quality of evidence, Higher-order aberrations (HOAs). Figure S8: quality of evidence (MTF cut-off). Figure S9: quality of evidence (Strehl ratio). [file 4728258.f1.zip › 4728258.f1/s7.jpeg]

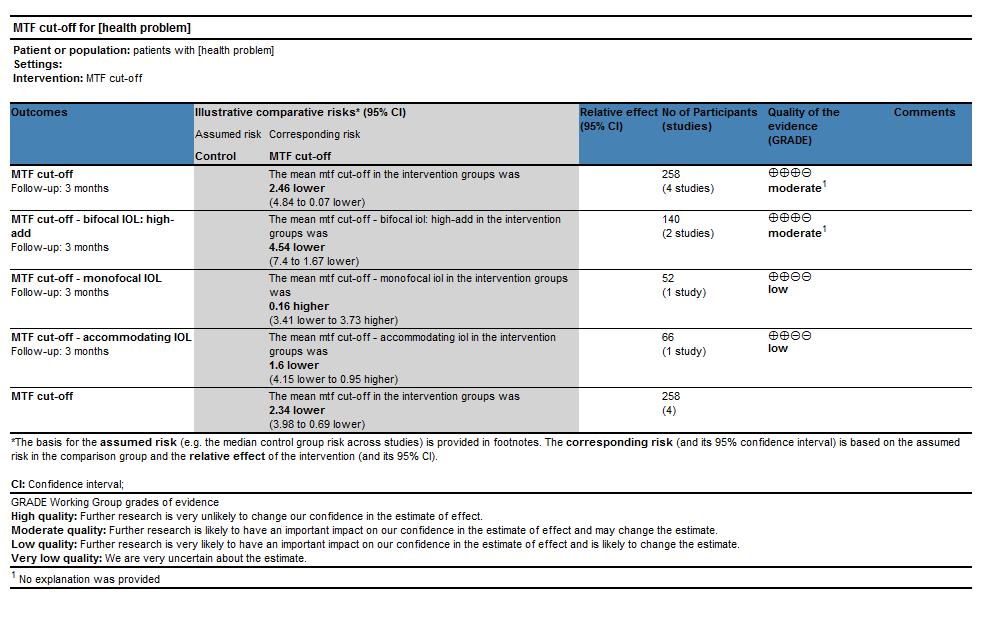

Supplement: Supplementary Materials — Figure S1: quality of evidence (UDVA). Figure S2: quality of evidence (CDVA). Figure S3: quality of evidence (UIVA). Figure S4: quality of evidence (UNVA). Figure S5: quality of evidence (DCNVA). Figure S6: quality of evidence (CNVA). Figure S7: quality of evidence, Higher-order aberrations (HOAs). Figure S8: quality of evidence (MTF cut-off). Figure S9: quality of evidence (Strehl ratio). [file 4728258.f1.zip › 4728258.f1/s8.jpeg]

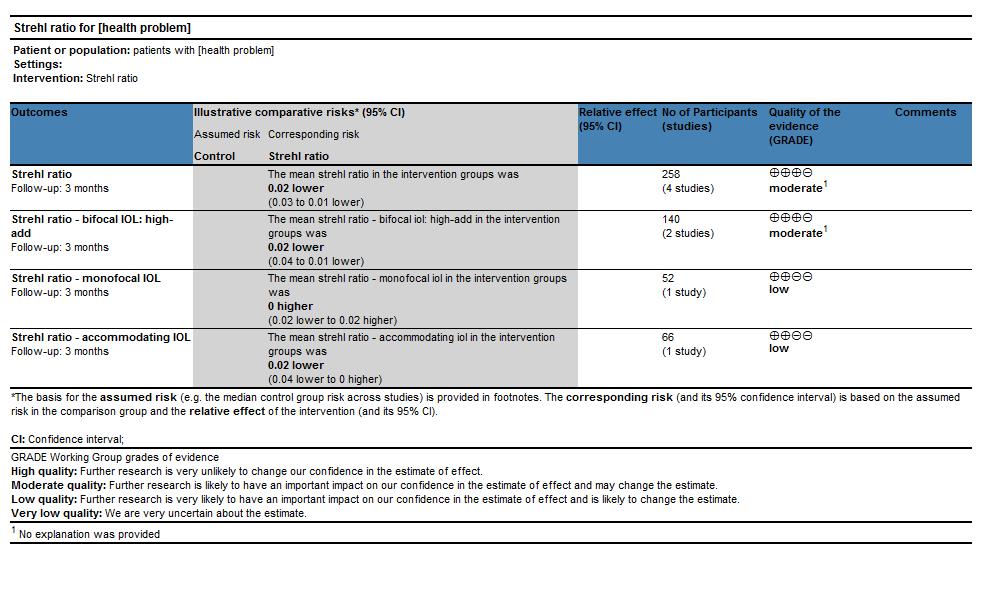

Supplement: Supplementary Materials — Figure S1: quality of evidence (UDVA). Figure S2: quality of evidence (CDVA). Figure S3: quality of evidence (UIVA). Figure S4: quality of evidence (UNVA). Figure S5: quality of evidence (DCNVA). Figure S6: quality of evidence (CNVA). Figure S7: quality of evidence, Higher-order aberrations (HOAs). Figure S8: quality of evidence (MTF cut-off). Figure S9: quality of evidence (Strehl ratio). [file 4728258.f1.zip › 4728258.f1/s9.jpeg]
